# Alteration of the IFN-Pathway by Human Papillomavirus Proteins: Antiviral Immune Response Evasion Mechanism

**DOI:** 10.3390/biomedicines10112965

**Published:** 2022-11-17

**Authors:** Leonardo Josué Castro-Muñoz, Leticia Rocha-Zavaleta, Marcela Lizano, Katia Montserrat Ramírez-Alcántara, Vicente Madrid-Marina, Joaquín Manzo-Merino

**Affiliations:** 1 The Wistar Institute, 3601 Spruce Street, Philadelphia, PA 19104, USA; 2 Unidad de Investigación Biomédica en Cáncer, Instituto Nacional de Cancerología, México/Instituto de Investigaciones Biomédicas, Universidad Nacional Autónoma de México, Av. San Fernando No. 22, Col. Sección XVI, Tlalpan, Mexico City 14080, Mexico; 3 Departamento de Biología Molecular y Biotecnología, Instituto de Investigaciones Biomédicas, Universidad Nacional Autónoma de México, Circuito Escolar S/N, Ciudad Universitaria, Delegación Coyoacán, Mexico City 04500, Mexico; 4 Departamento de Medicina Genómica y Toxicología Ambiental, Instituto de Investigaciones Biomédicas, Universidad Nacional Autónoma de México, Mexico City 04510, Mexico; 5 Dirección de Infecciones Crónicas y Cáncer, Centro de Investigación sobre Enfermedades Infecciosas (CISEI), Instituto Nacional de Salud Pública, Av. Universidad 655, Santa María Ahuacatitlán, Cuernavaca, Morelos 62100, Mexico; 6 Cátedras CONACyT-Instituto Nacional de Cancerología, San Fernando No. 22, Col. Sección XVI, Tlalpan, Mexico City 14080, Mexico

**Keywords:** HPV proteins, interferon-stimulated genes, immune response evasion

## Abstract

A persistent infection with the so-called high-risk Human Papillomaviruses (hr-HPVs) plays a fundamental role in the development of different neoplasms. The expression of the HPV proteins throughout the different steps of the viral life cycle produce a disruption of several cellular processes, including immune response, which can lead to cell transformation. The interferon-mediated response plays an important role in eliminating HPV-infected and -transformed cells. The ability of HPV to disrupt the proper function of the interferon response is based on a series of molecular mechanisms coordinated by HPV proteins intended to prevent clearance of infection, ultimately producing an immunotolerant environment that facilitates the establishment of persistence and cancer. In this review, we focus on the molecular actions performed by HPV E1, E2, E5, E6 and E7 proteins on IFN signaling elements and their contribution to the establishment of infection, viral persistence and the progression to cancer.

## 1. Introduction

Human Papillomavirus (HPV) infection is a highly prevalent sexually transmitted disease (STD) affecting a large proportion of the sexually active population worldwide [1]. According to different epidemiological studies, close to 90% of HPV infections are eliminated within two years due to an efficient immune response [2,3,4]. Nevertheless, a small proportion of such infections persist and eventually progress to premalignant lesions and, in exceptional cases, to cancer. Persistent infection with hr-HPV represents a necessary requisite for the development of different neoplasms [5]. A lot of evidence indicates that a failure in interferon (IFN) signaling prevents the elimination of infected cells, which strongly contributes to the establishment of cancer. This review describes the principal IFN-mediated mechanisms involved in the recognition and clearance of HPV infections as well as the different actions implemented by HPV proteins to inhibit such an important response.

## 2. Human Papillomavirus

Human Papillomaviruses are small non-enveloped DNA viruses with an icosahedral capsid with more than 200 HPV types identified so far (PaVE: Papillomavirus Episteme). HPVs are classified into five genera: Alpha, Beta, Gamma, Nu and Mu, where Alpha and Beta papillomaviruses are considered of medical relevance [6]. The so-called high-risk Human Papillomaviruses (hr-HPVs) are associated with the development of different anogenital neoplasms, including cervical, vulvar, anal and penile cancers, as well as a subset of head and neck cancers, mostly oropharyngeal squamous cell carcinoma (OPSCC), HPV16 and 18 being the most prevalent types [7].

HPVs infect the basal cells of different epithelia, such as the cervix, anus, and oropharynx, accessing the basal layer through microtraumas [8,9,10]. Once in the nucleus, the viral genome remains as an episome exhibiting minimal gene activity and maintaining a low copy number (20–100 per cell), guided by the viral E1 and E2 proteins. The productive infection takes place in the middle layers of the epithelium, producing the amplification of the viral genome induced by the different actions of the E6, E7, E1 and E2 early proteins. First, E7 and E6 promote the S phase and inhibit apoptosis, while E1 and E2 bind to the origin of replication on the HPV genome, allowing its maintenance and amplification during epithelium differentiation [11]. The E5 protein favors cellular proliferation through the recycling of growth factors, as well as the evasion of the immune response [12]. At late stages, the L1 and L2 proteins are expressed at the upper layers of the epithelium, leading to the construction of the viral capsid. Finally, the E4 protein promotes the collapse of cytokeratin filaments, favoring the release of the new viral particles [13]. In most cases, early HPV infection is efficiently detected by the host’s immune response, and consequently, infected cells are readily eliminated [6].

During genome amplification, HPV-infected cells are detected by different immune surveillance effectors, including Natural killer (NK), dendritic cells, macrophages and T and B lymphocytes [14]. Nonetheless, HPV has proved to be an excellent immune blocker; thus, persistent infections are a clear consequence during the natural history of disease. In this regard, viral persistence allows viral genome integration leading to cancer development due to the loss of E2 ORF (open reading frame); this in turn produces an overexpression of E6 and E7 oncogenes [15], triggering the cellular transformation process. Additionally, the E5 protein cooperates during the HPV-induced transformation process, due to its capacity to promote cell proliferation and a deregulation of the immune response [12].

Although E5, E6 and E7 actions represent the main promoter of HPV-driven effects, the other HPV early proteins exhibit different functions that might contribute to viral persistency. For instance, the E2 protein is required for viral amplification, but is also involved in producing oxidative stress and apoptosis. Furthermore, E2 is also found during early stages of carcinogenesis [16]. The E1 gene, on the other hand, is expressed not only during a productive infection, but also during cervical carcinogenesis, as demonstrated by the high levels of the E1 mRNA found along the different stages of cervical cancer progression [17], indicating that this protein could be somehow involved in the carcinogenic process. The above-mentioned proteins denote that even when the transforming activities are mainly driven by E6 and E7, these other HPV proteins could affect different cellular processes, potentially contributing to HPV-mediated immune evasion.

## 3. Immune Responses for HPV Clearance

**Figure 1:**
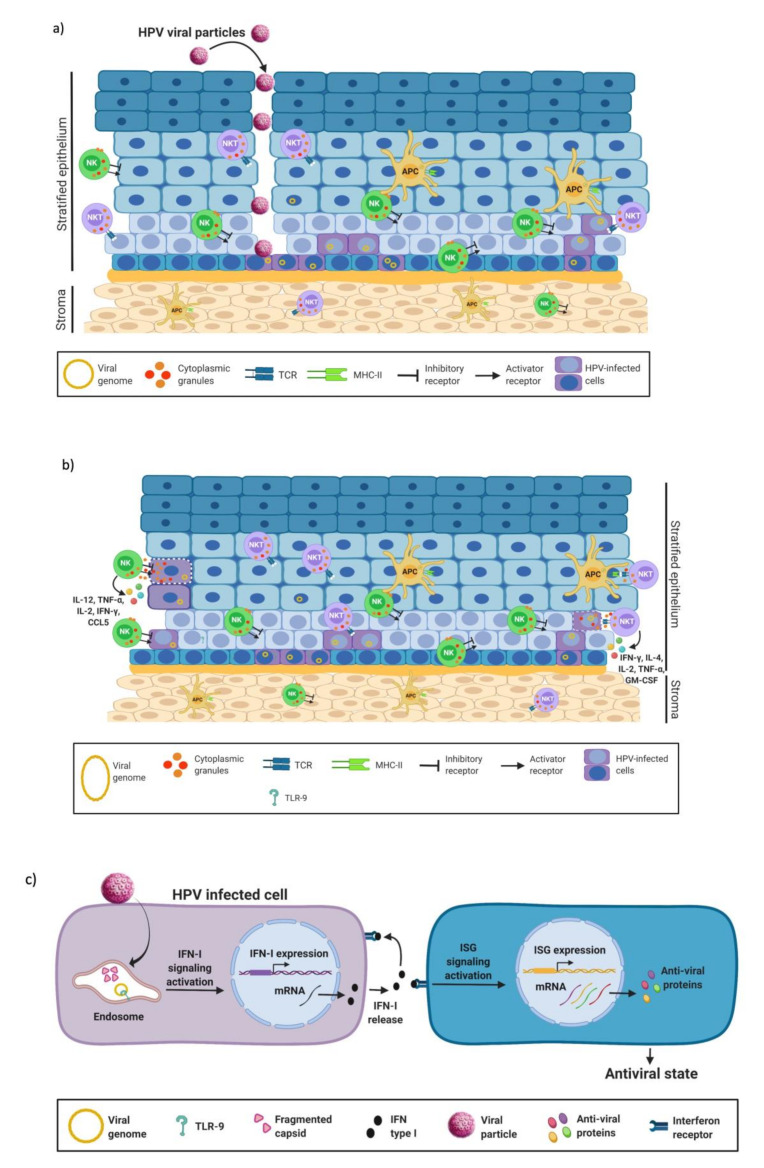
Innate immune response against HPV. (**a**) The HPV viral particle infects the basal cells, gaining access through microtrauma. Several elements of the immune response are highly active throughout the different layers of the epithelium including APC, NK and NKT, which are responsible for recognizing and clearing infectious agents. (**b**) Infected cells are recognized by NK cells through KIR receptors, inducing their elimination by releasing their cytoplasmic granules containing granzyme, perforin and cytokines. Moreover, NKT cells control infection by other mechanisms. For example, the CD1d receptor activates NKT cells to produce apoptosis of the infected cell and cytokine release that will help activate the adaptive immune response. NKT cells act as a bridge between innate and adaptive immunity. (**c**) Once the cell is infected by HPV, the TLR-9 receptor detects the viral genome in the cytoplasm. Then, TLR-9 activates IFN-I production and ISG expression promoting an antiviral effect. Figure created using BioRENDER.com.

The elimination of a viral infection depends mainly on the correct activation of the elements of the innate immune response, such as macrophages, polymorphonuclear cells (PMN), natural killer cells and natural killer T cells (NKT), which in turn give rise to a set of immunomodulatory molecules in the epithelium that help control the infectious process (Figure 1a) [14]. In addition, HPV-infected keratinocytes may act as non-professional antigen-presenting cells to promote the clearance of infected cells through the secretion of anti-viral and pro-inflammatory mediators [18,19].

NK and NKT cells are effector cells with an important role in the innate immune response against viruses. NK cells cause apoptosis and subsequent lysis of virus-infected or -transformed cells via their Killer-cell immunoglobulin-like receptors (KIR). Infected or transformed cells show decreased expression of MHC class I, which in turn triggers the activation of NK cells [20]. Once activated, NK cells release their cytoplasmic granular content which includes granzyme and perforin to cause cellular lysis and, in addition, yield IFN-γ and TNF-α [21]. The activation of the NK cells depends either on the action of MHC class I or on the functions of pro-inflammatory cytokines, such as IL-2, IL-12, IL-15 and IL-18, produced during a viral infection [22]. Moreover, NKT cells express a TCR composed of an invariant alpha chain, also called invariant NKT cells (iNKT) [23]. The iNKT cells detect exclusively glycolipids restricted to the CD1d receptor [24]. After viral infection, iNKT cells become activated and produce granzyme B and/or perforin, promoting the destruction of infected cells. Moreover, activated iNKT cells are able to produce a wide range of cytokines and chemokines, such as IL-2, IL-13, IL-17, IL-21, TNF-α, IL-4 and granulocyte-macrophage colony-stimulating factor (Figure 1b) [25].

Most cells mediating the innate immune response express pattern recognition receptors (PRRs) to detect different pathogens, including viruses. Among them, toll-like receptors (TLRs) are of particular interest since they recognize pathogen-associated molecular patterns (PAMPs), which are groups of chemical features common to certain types of pathogens [26]. TLRs are not only expressed by immune cells, but also by non-hematopoietic cells including keratinocytes, participating in the activation of the antiviral response [27]. TLR-9 recognizes non-methylated CpG sequences on the viral DNA; hence, this receptor is responsible for detecting the HPV genome at the cytoplasm [28]. Once activated, TLR-9 promotes the secretion of type I interferon (IFN-I), which is involved in the control of viral infection by inducing an antiviral cellular state (Figure 1c).

Another component of the innate immune response that favors IFN expression is cyclic guanosine monophosphate-adenosine monophosphate synthase (cGAS), which participates in the detection of cytoplasmic DNA, mostly from viral and bacterial pathogens. The recognition of foreign DNA by cGAS induces cGAMP synthesis which in turn binds to and activates STING at the ER membrane. Once activated, STING translocates to Golgi compartments, where it interacts with TBK1 or IκB kinase (IKK), an event that is facilitated by palmitoylation of STING. TBK1 phosphorylates STING, which in turn recruits IRF3 to be phosphorylated by TBK1. Phosphorylated IRF3 dimerizes and enters the nucleus, where it stimulates transcriptional expression of IFN-I [29,30].

## 4. The IFN-Pathway

**Figure 2:**
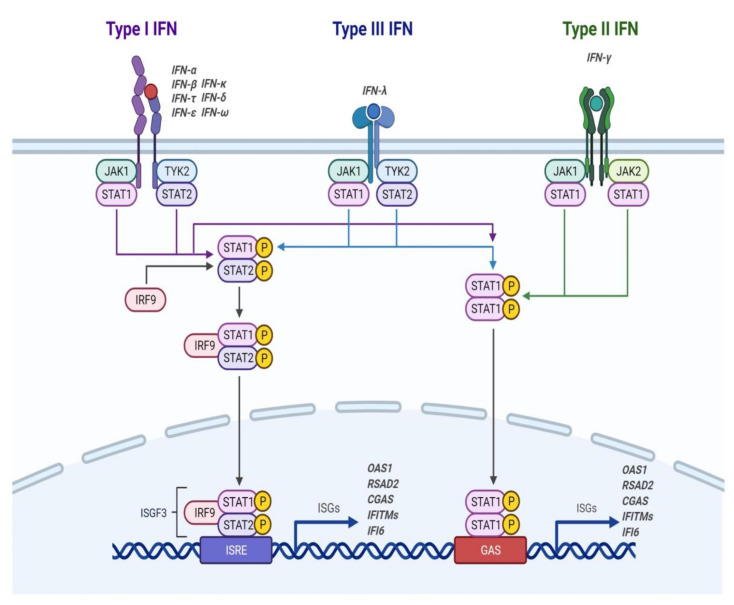
Activation of the IFN pathway. Within types I and III, there are multiple IFNs; within type II, there is a single IFN. Each type has a different heterodimeric cell surface receptor: type I IFNs bind to the IFNAR receptor complex (composed of IFNAR1 and IFNAR2); type III IFNs bind to the IFNLR receptor complex (composed of IFNLR1 and IL-10Rβ). Binding of IFN to its receptor complex results in cross-phosphorylation of JAK1 and TYK2 in the cytoplasmic domains of the receptor subunits. This triggers the phosphorylation of STAT1 and STAT2. After phosphorylation, STATs form various complexes that translocate to the nucleus, where they bind to IFN-stimulated response elements (ISREs) or gamma-activated sequences (GAS) on ISG promoters. Binding to these promoter elements results in the transcription of hundreds of genes involved in the antiviral response, including OAS1, -2, -3, RSAD2, CGAS, IFITMs (-1, -2, -3, -5) and IFI6, among others, which will control the infectious process. Figure created using BioRENDER.com.

IFNs belong to a family of inducible cytokines which promote the so-called “antiviral state” in infected cells as well as in neighboring cells by activating signaling pathways that result in the activation of interferon-stimulated genes (ISG) (Figure 2). To date, three IFN types have been identified (type I, II and III), of which I and III are involved in the innate immune response. Type I IFNs include IFN-α, IFN-β, IFN-ε, IFN-κ and IFN-ω; IFN-γ is only member of type II IFNs, while IFN-III includes IFN-λ1 (IL-29), IFN-λ2 (IL-28A), IFN-λ3 (IL-28B) and IFN-λ4 [31]. Once secreted, IFNs bind to their specific receptor on the surface of different cells. Type I IFNs bind to the Interferon-alpha/beta receptor (IFNAR) complex (composed of IFNAR1 and IFNAR2), while type III IFNs bind to the lambda Interferon receptor (IFNLR) complex (composed of IFNLR1 and IL-10Rβ) [32,33].

Upon binding of IFN-I or IFN-III to their respective receptor, the intracellular portion of the receptor activates the Janus tyrosine kinases (JAKs) that result in the subsequent phosphorylation of the signal transducer and activator of transcription (STAT) proteins. Consequently, phosphorylated STAT1 and STAT2 heterodimerize and interact with the IFN regulatory factor (IRF) 9, forming the ISG factor 3 complex (ISGF3). ISGF3 then translocates to the nucleus, where it binds to IFN-stimulated regulatory elements (ISRE) that promote the transcription of about 300 known ISGs [34]. These activated ISGs act at different levels of the viral replicative cycle aimed to control the infectious process.

The antiviral effects of IFN-I are mediated by the induction of several interferon-stimulated genes impairing viral replication through multiple mechanisms, such as the inhibition of protein translation and degradation of viral RNA. ISGs also activate and help with the survival of innate and adaptive immune cells including dendritic cells (DCs), macrophages, NK cells and T cells [35]. Therefore, IFN-I helps to control the infectious process and improves the adaptive immune response.

## 5. Deregulation of the IFN Pathway by HPV Proteins

**Figure 3:**
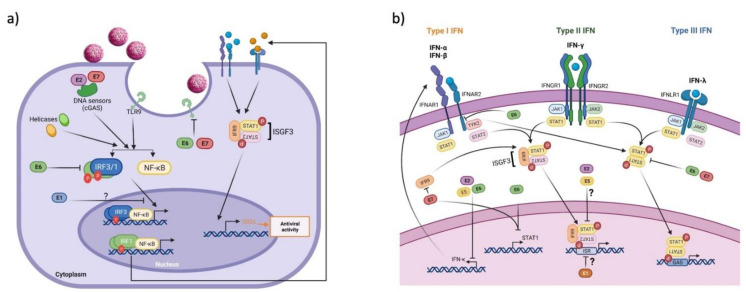
Effect of HPV proteins on IFN production and the ISG pathway. When the cell senses viral DNA, signaling pathways are triggered to produce IFN; then, the binding of IFN to its receptors activates the ISG pathway to control viral infection. (**a**) HPV proteins affect IFN production: E6 and E7 oncoproteins interact with TLR and cGAS receptors and also with transcriptional factors such as IRF-3 and -1 that block IFN production. In addition, E1 protein prevents the expression of IFN-β and IFN-λ; however, the mechanisms of such regulation are still unclear. (**b**) The production of IFN promotes the activation of the ISG pathway; however, HPV proteins prevent its activation by acting at various levels of regulation of the pathway. However, it is not known if the inhibition of IFN is enough to prevent the activation of ISGs or whether other components such as STAT1 and STAT2 could be regulated by these proteins. IFN, interferon; IFNAR, IFN-α receptor; IFNGR, IFN-γ receptor; IFNLR, IFN-λ receptor; IRF9, interferon regulatory factor 9; ISGF3, interferon stimulated gene factor 3; ISRE, interferon stimulated response element; GAS, gamma interferon activation site. Figure created using BioRENDER.com.

HPV induces a deregulation of several components of the immune response in the IFN pathway (innate response immunity) to produce a successful infection, complete the viral life cycle, allow for viral persistence and, in exceptional cases, lead to the development of cancer [36]. Next, we describe the main actions carried out by each of the HPV early proteins (E1, E2, E5, E6 and E7) in the deregulation of the antiviral immune response mediated by interferon. Specifically, how viral proteins alter interferon production (Figure 3a) and how they regulate the ISG pathway to prevent its activation (Figure 3b).

### 5.1. HPV E1 Protein

The early E1 protein has been mostly associated with viral replication and it is the only viral protein with enzymatic activity, necessary throughout the HPV replicative cycle [37]. A study carried out by Terenzi et al. (2008) showed the effect of the IFN and ISG on the HPV E1 protein. It showed that when p56, an interferon-induced protein, is overexpressed, it interacts and inhibits the helicase activity of HPV16 E1 [38]. However, recent studies show that E1 may affect different processes aimed at regulating the immune response. In this regard, Castillo et al. (2014) found that different set of genes are deregulated after silencing the expression of the E1 gene in HPV18 positive HeLa cells. Mostly, these differentially expressed genes were grouped into four categories including: TLR signaling pathway, IFN signaling pathway, antiviral ISG and apoptosis [39]. This indicates that E1 might impair proper functioning for the recognition and clearance of infected cells.

In addition, E1 from different HPV types was shown to downregulate genes involved in the antiviral defense including IFNβ1 and IFNλ1, as well as some IFN-stimulated genes (CCL5, Viperin and IFIT2) [40]. Interestingly, E1 proteins from different HPV types were also found to decrease the expression of IFNβ1 and IFNλ1, even after the addition of a potent stimulator of the IFN-β1 pathway (poly I:C). Therefore, the HPV E1 protein can inhibit the innate immune response; however, the mechanisms by which it affects the IFN pathway have not yet been investigated. One possibility could be associated with direct DNA binding, since E1 possesses such ability. On the other hand, E1 could be acting indirectly throughout its interaction with transcriptional regulators. Interestingly, E1 mRNA has been shown to increase with an increasing grade of cervical intraepithelial neoplasia (CIN), with higher levels found in cervical cancer [17,41]. This indicates that E1 could have effects beyond the viral genome amplification and that the inhibition of the immune response could be contributing to the carcinogenic process; however, this possible role of the E1 protein remains to be explored.

### 5.2. HPV E2 Protein

E2 is involved in a plethora of actions including initiation of DNA replication, cellular and viral transcriptional regulation and episomal segregation [42]. Interestingly, regulation of cellular gene expression has been proposed as a potential mechanism involved in viral pathogenesis, including the deregulation of genes involved in cell proliferation, apoptosis, differentiation and immune regulation [43,44,45].

Regarding the actions of E2 on the regulation of the immune response, there is little evidence showing that E2 protein can affect the innate immune response through the regulation of the IFN expression. Sunthamala et al. (2014) demonstrated a reduction in IFN-κ and STING mRNAs in primary keratinocytes expressing E2 from hr-HPVs even after stimulating IFN with poly I:C. Furthermore, they observed that the E2 transactivation domain was responsible for the suppression of IFN-κ and STING. Additionally, downregulation of STING and IFN-κ was also corroborated in clinical specimens in which these genes and some ISGs were downregulated in HPV-positive low-grade squamous intraepithelial lesions (LSIL) compared with HPV-negative controls [45]. These results provide clues about the possible role of E2 in the evasion of the innate immune response that could be an immune escape mechanism during the viral replication, persistence and development of HPV-associated cancer.

### 5.3. HPV E5 Protein

The HPV E5 protein has immunosuppressive functions that could favor the replicative cycle, but it has also shown transforming activity in cellular and animal models [46]. The participation of E5 in the immune response implies different actions in both the adaptive and innate responses. For example, Muto et al. (2011) showed that the HPV16 E5 protein increases IFN-β1 levels due to the increased expression of the IRF-1 transcription factor; in addition, they observed an increase in the expression of ISGs (PKR and caspase 8) [47]. These data could be considered controversial since E5 would be expected to inhibit IFN expression, favoring the replicative cycle. However, there is evidence that this could be a mechanism that favors carcinogenesis, since treatment with IFN in W12 cells (cervical epithelial cells that contain episomal copies of the HPV16 genome) causes a decrease in the number of viral copies which, in turn, causes an increase in the number of integrated genomes [48]. In addition, the E5 protein inhibits the expression of IFN-κ and some ISGs, which is dependent of TGF-β signaling. Interestingly, loss of E5 expression in HPV16-positive primary human foreskin keratinocytes (HFKs) increase IFN-κ levels and increase the number of viral integrated copies. Although it is unclear how IFN might be promoting viral genome integration, it is believed that it may be due to the ability of IFNs to deplete cells with episomal copies, allowing cells containing integrated copies to predominate. IFN promotion of viral genome integration could also be because the chronic expression of ISGs results in genomic instability and favors the integration of the HPV genome [49]. These data indicate that it is interesting not only to study how the HPV E5 protein can regulate the IFN signaling pathway to inhibit the innate immune response, but also how it could be favoring carcinogenesis.

### 5.4. HPV E6 and E7 Proteins

There is increasing evidence that the E6 and E7 proteins are capable of modulating the IFN-I signaling pathway. Nees et al. (2001) evaluated the differential expression of mRNA in primary keratinocytes expressing HPV16 E6 and E7 and found that 80 cellular genes were deregulated in the presence of the oncoproteins. These genes were grouped into three clusters: (1) interferon (IFN)-responsive genes; (2) genes stimulated by NF-κB; and (3) genes regulated in cell cycle progression and DNA synthesis [50]. These results indicated that HPV oncoproteins affect the immune response.

Individually, it has been shown that E6 and E7 oncoproteins influence the innate immune response, primarily by affecting interferon functions. E6 interacts with interferon regulatory factor 3 (IRF-3, a transcriptional factor for IFN-β), preventing its transactivation activity and consequently inducing its degradation via the proteasome system; this effect is associated with an increased viral replication capacity [51]. Additionally, E7 inhibits the translocation of the transcriptional factor p48, a component of the interferon-stimulated gene factor 3 (ISGF3) transcription complex (STAT1, STAT2 and p48) responsible for IFN-α production. This suggests that E7-mediated inhibition of p48 nuclear activity results in loss of IFN-α-mediated signal transduction, which may impair the action of the innate immune system [52].

Another important component of the innate immune response is the cGAS-STING pathway which favors the transcriptional expression of IFN-I to initiate the antiviral immune response [29]. The E7 oncoproteins of HPV16 and 18 have been shown to antagonize the cGAS-STING pathway by preventing IFN-β expression. This is due to the ability of E7 to interact with STING through the LXCXE motif (pRb binding motif), whereas E7 silencing in cell lines derived from HPV-positive cervical cancer and oropharyngeal squamous cell carcinomas restores the cGAS-STING pathway [53,54].

Furthermore, the E7 protein of HPV16 interacts with interferon regulatory factor 1 (IRF-1), a transcriptional factor required for the expression of IFN-β. E7 associates with the Nucleosome Remodeling Deacetylase (NURD) complex containing HDAC3, inducing transcriptional silencing of IRF-1-activated genes, including IFN-β [55]. HPV oncoproteins also affect other components of the IFN pathway, such as the IFN-α receptor. E6 prevents STAT2 and STAT1 phosphorylation by interacting with tyrosine kinase 2 (TYK2), thereby inhibiting ISG expression [56]. In addition, Cigno et al. (2020) found that HPV E7 proteins from types 16 and 18 promote the expression of the histone methyl transferase SUV39H1, fostering the transcriptional repression of RIG-I, cGAS and STING, while the inhibition of SUV39H1 leads to the transcriptional activation of these genes and increased production of IFN-β and IFN-λ HPV [57]. In this way, the E6 and E7 oncoproteins inhibit the signaling pathway mediated by IFN-I, hindering an important antiviral effector mechanism that has been shown to actively participate in the elimination of HPV.

Interestingly, keratinocytes, but not oropharyngeal epithelial cells, express IFN-κ, which belongs to human-type I IFNs and is constitutively expressed at detectable levels in uninfected cells. IFN-κ induces ISG transcription to control viral infections, representing a potential component to be disturbed during HPV infections and cancer [58,59]. Reiser et al. (2011) found that hr-HPV positive cells had reduced IFN-κ mRNA levels compared to normal keratinocytes. Furthermore, they demonstrated that HPV16 E6 shows a greater inhibitory effect on IFN-κ expression compared to E7 oncoprotein; nonetheless, both oncoproteins produce significant downregulation. The decrease of IFN-κ by E6 affects the expression of different ISGs such as IFIT1, MDA5, MX1, RIG-I, STAT1, TLR3, TRAIL and XAF1. The proposed mechanism by which E6 inhibits the expression of IFN-κ is through p53 degradation since recovery of p53 expression results in increased IFN-κ levels in cell lines derived from cervical cancer [60]. All these reports suggest that HPV E6 and E7 oncoproteins inhibit key components of the antiviral immune response, thus preventing the recognition of HPV in the early stages of infection and favoring the replicative cycle, persistence and development of cancer.

Another way that viruses affect IFN expression is through the inhibition of receptors that favor IFN expression [61]. Particularly, in the case of hr-HPV infections the E6 and E7 oncoproteins regulate a plethora of cellular factors involved in the regulation of cell proliferation, cell death and, importantly, host defense. In this sense, Hasan et al. (2007) demonstrated that HPV16 E6 and E7 oncoproteins block the activation of innate immunity by decreasing the levels of TLR-9 mRNA [62]. The authors also showed that both HPV16 E6 and E7 bind to the TLR9 promoter, preventing transcription of the TLR9 gene compared to low-risk HPVs. Similar results were observed in cervical cancer-derived cell lines, in which the TLR-9 expression was weaker in HPV16-positive SiHa cells and completely absent in Ca Ski cells. In contrast, HeLa cells that contained multiple copies of integrated HPV18 exhibited higher levels of TLR-9 mRNA, likely indicating a viral type-dependent effect. In addition, it was determined that HPV16 E7 also prevents the expression of TLR9 by forming a nuclear complex on the TLR9 promoter, composed by the estrogen receptor α (ERα), NF-κBp50 and p65. This in turn allows the recruitment of histone deacetylase (HDAC) 1 and histone demethylase JARID1B, which contributes to transcriptional silencing of TLR-9 in C33A cells [63]. Moreover, the authors evaluated the expression of NF-κBp65 and ERα, finding increased levels in HPV16-positive cervical cancer samples when compared to normal controls. These data support the claim that HPV E6 and E7 oncoproteins downregulate the expression of TLR-9 at a transcriptional level, affecting the recognition of viral DNA by the innate immune system and consequently blocking the production of IFN-I and pro-inflammatory cytokines.

In support of the downregulation of TLR-9 by HPV, a low expression of TLR-9 was found in HPV16-positive cervical biopsies compared to HPV-negative normal tissues [62], although several studies have demonstrated an increased expression of TLR-9 in cervical cancer samples of women with HPV infections, squamous intraepithelial cervical lesions and cancer [64,65,66,67]. Therefore, the actual status of TLR-9 in HPV-induced disease remains unclear and somehow controversial. Likewise, it has been observed that in HPV-positive oropharyngeal cancer the expression of TLR-9 is increased compared to HPV-negative counterparts, although a pro-inflammatory response has not been found after stimulation with LPS. Therefore, this indicates a differential behavior of the TLR-9 signaling in HPV-induced neoplasia [68]. Therefore, further studies are needed to clarify the role of TLR-9 in preventing HPV infection or promoting cancer development.

Certainly, the regulation of the IFN pathway involves different mechanisms that are targets of viral proteins. There is evidence supporting the claim that HPV E6 and E7 oncoproteins deregulate cellular miRNAs [69,70,71] and studies have focused on evaluating the consequences of such a deregulation on targets related to carcinogenesis. Although there are no direct studies indicating that HPV oncoproteins deregulate miRNAs related to the interferon pathway, it has been reported that miR-146a was constantly found upregulated in E5-expressing HaCaT [72] and it is known that miR-146a regulates the adaptive and innate immune responses [73]. Particularly, the production of IFN-gamma by natural killer cells is ablated by miR-146a via targeting NF-kB [74]. In the same sense, it has been reported that miR-146a is overexpressed in human keratinocytes expressing HPV16 E6 and E7 oncoproteins [69]; moreover, the authors also reported that miR-155 is downregulated in E6 expressing cells. Meanwhile, it has been demonstrated that the downregulation of miR-155 in NK-stimulated cells suppresses the production of IFN-gamma [75]. In such a way, indirect evidence suggests that E6, E7 and E5 oncoproteins modulate the expression of miRNAs, which can alter the IFN pathway.

## 6. Concluding Remarks

An important factor for viral replication and cancer progression is the failure of the immune system. The coordinated actions of elements of the innate and adaptive immune responses achieve the elimination of both infected cells and transformed cells. The IFN signaling pathway plays an important role within the innate immune response to control HPV infections. However, HPV early proteins can act on different mechanisms aimed to prevent the action of the IFNs and ISGs, allowing the establishment of a persistent infection and progression to cancer. Although many studies have focused on the E6 and E7 oncoproteins and their effects on IFN signaling, it is beginning to be understood how other HPV early-expressed proteins, such as E1, E2 and E5, can regulate IFN, favoring viral persistence and/or carcinogenesis. Thus, a knowledge of the specific molecules and cells that participate in the detection and elimination of cells infected or transformed by HPV, as well as an understanding of the mechanisms by which HPV proteins elude the immune system, will allow the design of new strategies aimed at enhancing the immune response against HPV. This will ultimately have an impact on clearing HPV-related premalignant lesions and assist in cancer prevention.

## Data Availability

Not applicable.

## Abbreviations

HPV: Human Papillomavirus; STD, sexually transmitted disease; hr-HPV, high-risk Human Papillomavirus; bp, base pairs; LCR, long control region; PaVE, Papillomavirus Episteme; LR-HPV, low-risk Human Papillomavirus; RRP, recurrent respiratory papillomatosis; ORF, open reading frame; E6AP, E6 associated protein; TNFR1, tumor necrosis receptor 1; ER, endoplasmic reticulum; EGFR, epidermal growth factor receptor; EGF, epidermal growth factor; COX-2, ciclioxygenase-2; VEGF, vascular endothelial growth factor; KGFR/FGFR2b, keratinocyte growth factor receptor/fibroblast growth factor receptor 2b; PMN, polymorphonuclear cells; NK, natural killer cells; NKT, natural killer T cells; HD5, human alpha defensin 5; PRRs, pattern recognition receptors; TLR, toll like receptor; PAMPs, molecular patterns associated with pathogens; IFN-I, interferon type I; MHC-I/II, major histocompatibility complex class I/II; ISG, interferon-stimulated genes; DCs, Dendritic cells; KIR, Killer-cell immunoglobulin-like receptors; TNF-α, Tumor necrosis factor α; IL, interleukin; iNKT, invariant NKT cells; APC, antigen-presenting cell; Th1/2, T helper cells 1/2; TCR, T- cell receptor; CTL, cytotoxic T cells; FasL/FasR, Fas ligand/Fas receptor; li, invariant chain; CLIP, small fragment of li; HLA-DM, leukocyte antigen-DM; v-ATPase, vacuolar H^+^-ATPase, TGF-β, Transforming growth factor β; mRNA, messenger ribonucleic acid; ERα, estrogen receptor α; HDAC, Histone Deacetylase; IRF, interferon regulatory factor; ISGF3, interferon-stimulated gene factor 3; NURD, Nucleosome Remodeling Deacetylase; TAP, Transporter Associated with antigen Processing protein.
